# Antimicrobial Stewardship in Non-Traditional Settings

**DOI:** 10.3201/eid3006.240255

**Published:** 2024-06

**Authors:** Nehal Hashem, Christina Yek

**Affiliations:** National Institutes of Health, Bethesda, Maryland, USA

**Keywords:** Antimicrobial resistance, stewardship, Centers for Disease Control and Prevention, guidelines, book review

In 2014, the US Centers for Disease Control and Prevention launched a new set of core elements for acute-care hospitals to implement key strategies in antimicrobial stewardship (AS). Over the following years, those guidelines were gradually expanded to include outpatient spaces and long-term care facilities. However, uptake in these areas has remained sluggish in the United States, in part due to limited resource allocation, lack of supporting evidence, and competing healthcare priorities. Antimicrobial Stewardship in Non-Traditional Settings offers a timely and comprehensive examination of AS implementation strategies and potential pitfalls, emphasizing the importance and feasibility of AS beyond the confines of acute-care settings (Figure).

**Figure:**
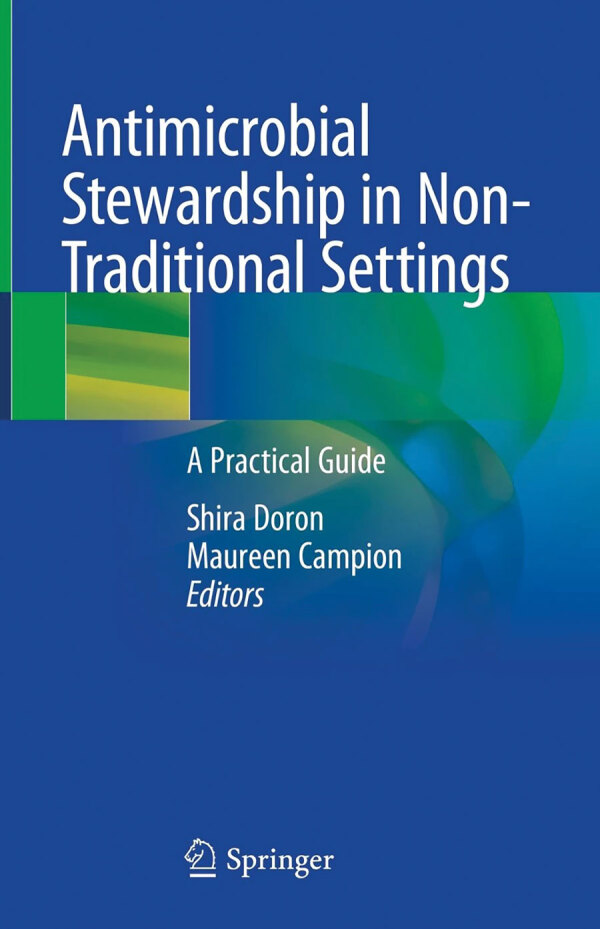
Antimicrobial Stewardship in Non-Traditional Settings

The book begins by highlighting the evolution of AS initiatives. Each subsequent chapter focuses on a distinct setting (e.g., long-term care facilities, ambulatory surgery centers, veterinary practices) or population (immunocompromised patients, pediatrics). The authors provide targeted strategies and practical recommendations for initiating AS programs and address the unique challenges encountered in each environment.

The discussion of antimicrobial resistance in long-term care facilities in the United States provides perhaps the clearest picture of how guidance from the CDC and other professional societies can be applied. Implementing such guidelines in other settings remains a less precise practice, and it is in discussion of these settings that the book truly shines. For instance, in discussing outpatient clinics and emergency departments, the authors perceptively note predominant barriers to effective AS to be improper documentation, challenges in formulating local antibiograms, and inadequate standardization of antibiotic use across facilities. The authors propose several solutions: use of rapid diagnostics, implementation of AS-friendly order-sets, and inclusion of multidisciplinary teams to strengthen early recognition and intervention.

In chapter 5, the authors describe how ambulatory surgery centers, often the more lucrative divisions within healthcare facilities, have increased incentives to reduce antimicrobial resistance and associated postsurgical complications. They propose that initiatives set forth in such well-funded facilities (e.g., standardized approaches for wound classification, decolonization practices) offer a unique template for implementing similar AS initiatives in less-resourced areas, such as small-access hospitals and veterinary practices.

In the last chapters of the book, the authors discuss an imbalance regarding the specific niche populations on which AS programs are focused nationwide, noting that patients in intensive care units are among the groups most targeted. Conversely, immunocompromised and pediatric patients remain understudied and underserved. The authors draw examples from outside the United States to support potential solutions for immunocompromised patients, but those suggestions are somewhat limited in the absence of evidence-based best practices. 

Antimicrobial Stewardship in Non-Traditional Settings offers a comprehensive and insightful exploration of AS implementation beyond acute-care settings and serves as a call-to-arms for medical and scientific communities tasked with exploring AS solutions for settings and niche populations most in need of such guidance. This book serves as a valuable resource for healthcare professionals striving to combat antimicrobial resistance, providing them with targeted strategies for diverse healthcare environments. The book’s breadth of coverage and practical recommendations make it an indispensable tool for advancing AS initiatives across various settings.

